# Papillomaviruses Go Retro

**DOI:** 10.3390/pathogens9040267

**Published:** 2020-04-07

**Authors:** Jian Xie, Pengwei Zhang, Mac Crite, Daniel DiMaio

**Affiliations:** 1 Department of Genetics, Yale School of Medicine, P.O. Box 208005, New Haven, CT 06520-8005, USA; jian.xie@yale.edu (J.X.); pengwei.zhang@yale.edu (P.Z.); 2 Department of Microbial Pathogenesis, Yale School of Medicine, 295 Congress Avenue, New Haven, CT 06519, USA; mac.crite@yale.edu; 3 Department of Therapeutic Radiology, Yale School of Medicine, P.O. Box 208040, New Haven, CT 06520-8040, USA; 4 Department of Molecular Biophysics & Biochemistry, Yale University, P.O. Box 208024, New Haven, CT 06520-8024, USA; 5 Yale Cancer Center, P.O. Box 208028, New Haven, CT 06520-8028, USA

**Keywords:** HPV, papillomavirus, retromer, cell-penetrating peptide, retrograde trafficking, virus entry, cervical cancer, endosome, γ-secretase

## Abstract

Human papillomaviruses are important pathogens responsible for approximately 5% of cancer as well as other important human diseases, but many aspects of the papillomavirus life cycle are poorly understood. To undergo genome replication, HPV DNA must traffic from the cell surface to the nucleus. Recent findings have revolutionized our understanding of HPV entry, showing that it requires numerous cellular proteins and proceeds via a series of intracellular membrane-bound vesicles that comprise the retrograde transport pathway. This paper reviews the evidence supporting this unique entry mechanism with a focus on the crucial step by which the incoming virus particle is transferred from the endosome into the retrograde pathway. This new understanding provides novel insights into basic cellular biology and suggests novel rational approaches to inhibit HPV infection.

## 1. Introduction

To reach the site of viral genome replication, most DNA viruses journey from the cell surface to the nucleus. This review summaries the unique entry mechanism used by papillomaviruses, with a focus on recent results that have clarified several key aspects of intracellular HPV trafficking during virus entry, and suggests potential therapeutic approaches. For comprehensive reviews on HPV entry, readers are referred to the excellent reviews by Campos, Sapp, and Banks and their colleagues [1,2,3].

The non-enveloped papillomavirus particle contains only two viral proteins, the major capsid protein L1 and minor capsid protein L2. Seventy-two pentamers of the L1 protein form the outer shell of the capsid, which also contains 12 to 72 molecules of the L2 protein and the ~8000 base pair double-stranded DNA viral genome bound to nucleosomes [4]. The L1 protein binds to heparan sulfate proteoglycans on the cell surface to initiate infection, while the L2 protein plays crucial roles in proper trafficking of the viral DNA to the nucleus and, in some HPV types, in packaging the viral DNA during virion assembly [1,5,6,7]. After initial cell binding, the virus particle undergoes a series of conformational changes and proteolytic cleavage events before it is internalized via a poorly understood but apparently unconventional endocytosis mechanism [8,9,10,11]. Most analyses of these and subsequent events during cell entry use pseudoviruses that encapsidate a reporter plasmid rather than the native viral genome [12]. Advantages of the pseudovirus system to study HPV entry include the non-pathogenic nature of the pseudovirus particle, the ease of producing large amounts of particles with wild-type or mutant capsid proteins, and the convenience of assaying successful infection by measuring reporter gene expression. Nevertheless, authentic papillomaviruses should be used to validate crucial findings obtained with pseudoviruses because there may be important differences between authentic viruses produced in stratified epithelial cells and pseudoviruses produced in a monolayer cell culture [13].

## 2. Role of Retrograde Trafficking and Retromer in HPV Entry

Knockdown and inhibitor studies have identified numerous cell proteins required for HPV entry and trafficking, including but not limited to furin, kallikrein-8, ADAM17, cyclophilin B, SNX17, SNX27, γ-secretase, VPS4, obscurin-like1, TSG101,TRAPPC8, growth factor receptors, DCT, annexin A2/S100A10, and tetraspanins CD131 and CD63 [8,10,14,15,16,17,18,19,20,21,22,23,24,25,26,27,28,29]. The proteases furin and kallikrein-8 cleave L2 and L1, respectively. Furin-catalyzed cleavage near the N terminus of L2 occurs at the cell surface, but in the absence of cleavage, intracellular trafficking events are disrupted [30]. ADAM17 appears to assemble an entry platform containing CDC151 and an epidermal growth factor receptor that mediates internalization of HPV [27]. In most cases, however, little is known about the mechanism by which these proteins support HPV infection. 

To comprehensively identify cellular proteins involved in HPV entry, we conducted a genome-wide siRNA screen. In this screen, ~20,000 different siRNA pools were transfected into HeLa cells in a microtiter plate format to repress individual cellular genes, and the ability of an HPV16 pseudovirus to express GFP from the encapsidated reporter plasmid was measured in each well of cells [31]. The results showed that efficient HPV infection requires numerous cellular proteins localized to vesicular compartments such as the trans-Golgi network (TGN) and the endoplasmic reticulum (ER). Subsequent validation studies and more targeted analysis of candidate genes confirmed that these proteins were required for HPV entry [30,31]. Among the required factors identified in these experiments were Rab proteins, which regulate vesicular transport [32,33], and retromer, a cellular trafficking machine that transports cellular proteins and toxins from the endosome to the TGN in a retrograde fashion [34]. These results raised the possibility that HPV itself may travel through the retrograde transport pathway to sites deep inside the cell. An alternative explanation is that a cellular protein essential for infection requires retrograde transport. An independent siRNA screen focused on later events in HPV entry and uncovered a requirement for nuclear envelop breakdown for nuclear import of incoming HPV [35].

These genetic findings inspired a series of immunodetection experiments that detected incoming HPV pseudovirus in intracellular compartments comprising the retrograde pathway [30,31]. In addition to standard immunofluorescence staining, proximity ligation assays were used in which a fluorescent signal is generated only if a viral protein is near to or in the same compartment as a cellular marker protein confined to that compartment [36,37]. Time course experiments revealed that during entry, viral capsid proteins are localized in the endosome by eight hours after infection, but at later time points depart from the endosome and appear in the TGN and Golgi apparatus. Indeed, HPV appears to reside inside membrane-bound compartments throughout the entry process until the onset of mitosis, and vesicles containing virus particles have even been visualized by electron microscopy in the nucleus [38,39,40]. By traveling to the nucleus inside vesicles, incoming HPV is likely to evade cytoplasmic innate immune sensors. Consistent with this prediction, HPV induces a weak interferon response in cultures of infected cells during virus entry [41].

**Figure 1:**
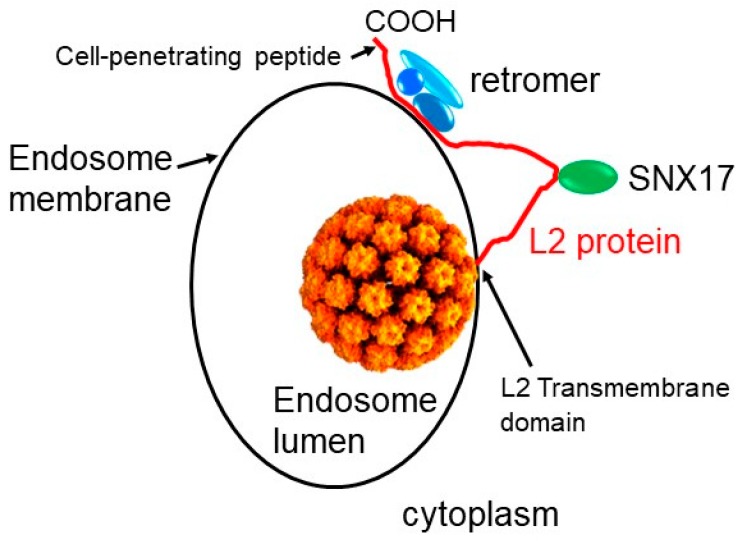
Schematic diagram of L2 protruding through the endosomal membrane into the cytoplasm. The papillomavirus capsid is shown in orange. The L2 protein is shown in red, bound to the retromer (blue) and SNX17 (green). COOH indicates the C terminus of L2. It has not been established how many amino acids of L2 protrude through the endosomal membrane, nor is it known if more than one L2 protein per capsid protrudes.

One of the key factors required for HPV entry is retromer, a cytoplasmic protein complex comprised of three core subunits, VPS26, VPS29, and VPS35 (Figure 1) [34]. The cargo selective core of retromer normally binds to the cytoplasmic domain of cellular transmembrane proteins that span the endosomal membrane. In conjunction with other cellular proteins, retromer causes the formation of tubules and vesicles containing membrane-bound cargo. After retromer dissociates from the cargo and the membrane, these vesicles traffic to the cell surface or the TGN where they fuse with target membranes to deliver their cargo. The normal function of retromer in retrograde transport suggested that retromer might deliver the incoming HPV particle into the retrograde pathway for trafficking to the TGN and beyond. This model that HPV is a retromer cargo is strongly supported by the finding that repression of retromer expression causes accumulation of incoming HPV in the endosome and its absence from downstream retrograde compartments, such as the TGN, Golgi apparatus, and ER [31,42]. 

**Figure 2:**
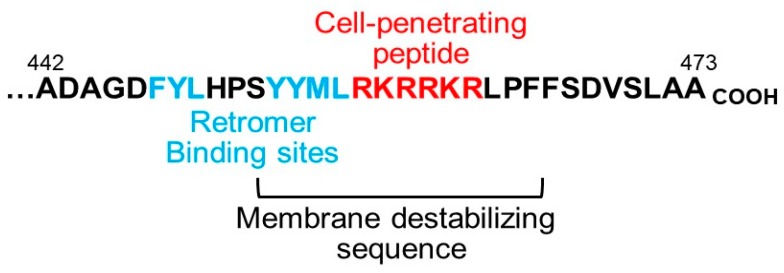
Essential sequence elements in the C terminus of the L2 protein. The C-terminal sequence of the HPV16 L2 protein is shown with the retromer binding sites, the cell-penetrating peptide, and the approximate extent of the membrane-destabilizing sequence indicated [42,44,45]. Of the two retromer binding sites, phenylalanine-tyrosine-leucine (FYL) is better conserved and more important in HPV entry. The numbers show the amino acid in L2.

Inspection of the amino acid sequence of the HPV capsid proteins identified a short hydrophobic sequence near the C terminus of the L2 protein, phenylalanine-tyrosine-leucine (FYL), that is conserved in all HPV L2 proteins and resembles a consensus retromer binding motif found in some cellular retromer cargos (Figure 2) [42,43].

Pull-down experiments with biotinylated peptides and recombinant fusion proteins purified from bacteria demonstrated that the L2 FYL sequence (as well as a nearby non-conserved YYML hydrophobic sequence) does in fact bind directly to retromer [42]. Importantly, studies with mutant pseudoviruses showed that these retromer binding sites in L2 are required for successful infection and that in the absence of these sites, exit of HPV from the endosome is blocked. This results in accumulation of the incoming virus in the endosome and its absence from distal compartments, the same phenotype caused by retromer knockdown. These experiments established that the retromer–L2 interaction delivers HPV into the retrograde pathway. Because the intact non-enveloped HPV capsid does not contain any transmembrane proteins, HPV is a novel form of retromer cargo unlike all other known cargoes, which are cellular transmembrane proteins. The inability of an entry-defective L2 mutant to escape the TGN is consistent with an important role of L2 in the transit of HPV through retrograde compartments during virus entry [46]. In addition to retromer, a related sorting complex known as retriever is also required for HPV entry, but retriever primarily transports proteins to the cell surface and the mechanism of retriever action in HPV entry is not known [47].

All HPV and most animal papillomaviruses contain FYL or a similar sequence near the C terminus end of L2, implying that retromer-mediated intracellular trafficking arose early in evolution of this virus family. However, a few animal papillomaviruses deeply rooted in the papillomavirus phylogenetic tree do not contain an obvious retromer binding site. It is not known if these viruses have a retromer binding site that is unrecognizable or if they utilize a different entry mechanism. 

Retromer function is normally regulated by various cellular proteins including sorting nexins and the Rab7 small GTPases [48,49]. Knockdown studies have shown that Rab7 is required for HPV entry, but the mechanism by which Rab7 supports HPV entry is not known [30,31]. In an unpublished protein interference screen, we identified the Rab7 GTPase-activating protein, TBC1D5, as a required HPV entry factor that supports HPV entry by regulating Rab7 and hence retromer action on incoming HPV (unpublished and [48]). In fact, Rab7 or TBC1D5 knockdown caused the HPV endosome accumulation phenotype characteristic of a disrupted L2–retromer interaction. Further experiments with dominant-negative and constitutively active Rab7 mutants showed that cycling of Rab7 between its GTP- and GDP-bound forms is required for retromer dissociation from HPV and for HPV endosome exit. As well as establishing the role of Rab7 in HPV entry, these results also imply that retromer-mediated HPV exit occurs from the late endosome where Rab7 is primarily localized. 

## 3. How HPV Fishes in the Cytoplasm for Entry Factors

The identification of HPV as a direct cargo of retromer poses a conundrum, because the endocytosed virus is in the lumen of the endosome, separated from retromer by the endosomal membrane. Several lines of published information suggest a possible resolution of this challenge. Sapp and colleagues reported that the C terminus of L2 contains a “membrane destabilizing sequence” required for HPV entry (Figure 2), and Campos and colleagues described the existence of a conserved hydrophobic, glycine-rich segment in the N-terminal portion of the L2 protein that can function as a transmembrane domain [44,50]. Finally, proteolytic cleavage experiments and immunostaining of selectively permeabilized cells suggested that much of the L2 protein is exposed in the cytoplasm during virus entry [51]. Taken together, these experiments suggested that during entry the C terminus of the L2 protein protrudes through the endosomal membrane into the cytoplasm, perhaps anchored in the membrane by a transmembrane domain at the other end of the protein (Figure 1). Once in the cytoplasm, L2 is in position to bind to retromer and other cytoplasmic entry factors such as SNX17 and SNX27 [15,21]

Protrusion of L2 through the endosome membrane is an attractive solution to the problem posed by the localization of the capsid and retromer in different cellular compartments, but insertion of proteins through membranes poses a significant energy barrier. In fact, most transmembrane proteins are inserted into membranes during translation via interactions between an N-terminal signal peptide and the complex translocation machinery at the ER membrane. In contrast, HPV is a non-enveloped virus particle lacking membranes or classic transmembrane proteins and enters cells as a virus particle comprised solely of mature soluble proteins. To determine if the C terminus of L2 actually extends through the endosome membrane into the cytoplasm, a split green fluorescent protein (GFP) assay was performed in which an inactive segment of GFP known as GFP11 was fused to the C terminus of the L2 protein, and the rest of GFP (GFP1-10) was expressed in the cytoplasm [45]. The two GFP segments can associate, reconstitute intact GFP, and generate a fluorescent signal only if they are both in the same cellular compartment [52]. Imaging studies of cells expressing GFP1-10 and infected with HPV16 pseudovirus containing GFP11-tagged L2 showed reconstituted fluorescence in the first few hours after infection, consistent with membrane protrusion of L2 into the cytoplasm early during the entry process. 

Although these experiments established that L2 protrudes into the cytoplasm, they did not address the mechanism of protrusion. The C-terminal segment of L2 near the retromer binding sites contains a highly conserved sequence of basic amino acids that resembles a class of protein segments known as cationic cell-penetrating peptides (CPPs) (Figure 2) [53]. CPPs were first discovered more than thirty years ago in the HIV Tat transactivator protein as a short amino acid sequence that could translocate the protein across the plasma membrane into cells [54]. Remarkably, CPPs from Tat and other sources can also translocate a variety of linked protein sequences and other elements into cells. However, the mechanism of membrane translocation is poorly understood, and although CPPs have received considerable attention as potential tools to deliver therapeutic molecules into cells, they have not entered clinical use.

Based on the sequence similarity between the basic segment of L2 and cationic CPPs, we hypothesized that the L2 basic segment might act as a CPP to deliver the L2 protein not across the plasma membrane but rather across the endosome membrane into the cytoplasm so that it could bind retromer and other HPV entry factors. Standard membrane translocation assays with the L2 basic sequence as a free-standing peptide or fused to GFP showed that it indeed has cell penetrating activity [45]. Mutant HPV16 pseudoviruses containing mutations that disrupt the CPP sequence are defective for virus entry and accumulate in the endosome, as is the case when retromer is knocked down or the retromer binding sites are mutated. These defects are rescued by replacing the L2 CPP with the CPP from HIV tat. Furthermore, split-GFP assays in infected cells show that, as predicted, mutations in the L2 CPP prevent membrane protrusion. Taken together, these experiments showed that a CPP at the C terminus of L2 mediates protrusion of the protein through the endosome membrane into the cytoplasm during entry so that it can directly engage retromer, which delivers HPV into the retrograde transport pathway ( Figure 1; Figure 2).

## 4. Implications for the Biology and Use of Cell-Penetrating Peptides

The experiments summarized above provide important new insights into not only the HPV entry process, but also cell biology in general. The biological roles of CPPs have been largely a mystery for decades. Because most prior studies focused exclusively on searching (unsuccessfully) for natural examples in which CPPs transferred secreted proteins across the plasma membrane into cells, other activities of CPPs may have been missed. In contrast to this supposed canonical CPP activity, the HPV CPP performs a very different role. Namely, it transfers a segment of a protein from one intracellular compartment into another. In addition, after membrane passage, the L2 N-terminal transmembrane domain may anchor L2 in the endosome membrane, so that the L2 protein adopts a transmembrane existence (Figure 1). Thus, another unanticipated activity of CPPs may be to convert soluble proteins into transmembrane proteins. We note, however, that the arrangement of the L2 protein after protrusion has not been firmly established.

Most CPPs studied to date are either artificial sequences or are excised from larger proteins with no known natural cell-penetrating activity. We speculate that many of these sequences display cell-penetrating behavior because of their underlying chemistry but in fact do not necessarily serve this function in vivo when linked to their host proteins. If this is the case, these sequences have not been selected by evolution for membrane passage activity. In contrast, all of the more than 300 sequenced papillomaviruses of both humans and animals have a C-terminal, basic L2 segment that presumably acts as a CPP during virus entry. Thus, this viral membrane-penetration activity has persisted for hundreds of millions of years of papillomavirus evolution. CPPs in extant L2 proteins therefore represent the results of the long evolutionary history of this virus group. Because they act in nature to transfer protein segments across membranes, papillomavirus CPPs are professional CPPs, in contrast to most CPPs studied to date, which may be amateurs. We also note that the rate-limiting step for CPP action in studied cases is not cell uptake but endosomal escape, precisely the natural activity of the papillomavirus CPPs. 

Although all papillomavirus CPPs are rich in basic amino acids, the precise sequences differ among different virus types. The CPP sequences found in multiple papillomavirus types (such as the CPP in HPV16, which is present in 16 different papillomaviruses) may be more active or have other favorable properties compared to CPPs present in only one or a few papillomavirus types. Similarly, the sequences flanking the core basic CPP, which are conserved but not identical among different papillomavirus types, may have been selected to enhance or fine-tune the activity of their associated CPP, for example to set the preferred pH or temperature of membrane protrusion. Continued analysis of papillomavirus CPPs and their flanking sequences will determine whether they are optimal for cargo translocation for practical uses. 

## 5. What Is the Trigger for Membrane Protrusion?

Papillomavirus particles are stable outside of their host cell. In the virus particle, the L2 protein is thought to be largely buried under the shell of L1 pentamers, but there is evidence that both the N terminus and C terminus of L2 are accessible on the surface of the particle before it encounters cells or soon thereafter [10,55,56]. Biochemical studies of purified recombinant L2 protein suggest that it is largely unstructured [57]. Membrane protrusion is presumably accompanied by a massive conformational change in the L2 protein and requires a considerable amount of energy. What triggers membrane protrusion after the virus enters the endosome? By analogy with the fusion proteins of enveloped viruses, which are triggered to insert short hydrophobic protein segments into (but not through) membranes, several potential triggers can be imagined: low pH caused by the influx of protons into the endosome lumen during endosome maturation; binding to other proteins, membranes, or small molecules; proteolytic cleavage or other post-translational modification of L2 or L1; or some combination of these or other factors. Endosome acidification is required for the early steps of HPV entry (and for the activity of the membrane destabilizing sequence that partially overlaps with the CPP) [44,58], so this might be a trigger for membrane protrusion. However, the precise entry step affected by acidification inhibition has not been established.

Papillomavirus entry is blocked by pharmacologic inhibition of the cellular protease γ-secretase or by knockdown of any of the four γ-secretase subunits, all of which are transmembrane proteins [23,24,31]. When γ-secretase is inhibited or repressed, HPV enters cells and localizes to the endosome at early times, but does not arrive at the TGN [56]. Unlike the situation when the binding of L2 to retromer is disrupted, the virus does not accumulate in the endosome in cells lacking γ-secretase activity, indicating that retromer and γ-secretase act at different steps in the HPV entry pathway. Biochemical studies show that γ-secretase binds directly to the N-terminal segment of the L2 protein and inefficiently cleaves L2 in or around its putative transmembrane domain [59]. Surprisingly, however, genetic reconstitution experiments in cells knocked out for the γ-secretase subunit PS1 revealed that the proteolytic activity of γ-secretase is not required for infection. Rather, γ-secretase displays a novel membrane chaperone activity that mediates insertion of L2 into the endosome membrane during HPV entry [59]. In these experiments, membrane insertion was assayed by preparing membranes from mechanically disrupted infected cells and then determining whether L2 was resistant to extraction by a high pH carbonate buffer. It will be interesting to determine if γ-secretase plays a similar non-canonical role in other biological processes that up to now have been assumed to require its protease activity. The vacuolar-ATPase inhibitor Bafilomycin A1, which inhibits endosome acidification, also inhibits L2 membrane insertion without blocking binding of L2 to γ-secretase, consistent with the model that low pH is a trigger for membrane insertion. The relationship between γ-secretase action, endosome acidification, membrane insertion, membrane protrusion, and retromer binding and action is undoubtedly complex and demands further investigation.

## 6. From Mechanism to Potential Therapeutics

Prophylactic vaccination will be the mainstay of HPV prevention efforts in coming years, but there is still need for alternative preventive approaches, because most people are not vaccinated and many pathogenic types of HPV (primarily cutaneous types) are not in current vaccine formulations. Can our newfound understanding of HPV entry suggest potential anti-viral strategies?

The retromer-mediated model of HPV retrograde entry suggests that agents that bind retromer would titrate retromer away from the virus and inhibit infection. Accordingly, we hypothesized that flooding the cytoplasm with peptides containing retromer binding sites would compete with L2 in the incoming virion for retromer binding and thereby inhibit HPV trafficking. As noted above, the limitation with most peptide therapeutics is inefficient delivery of the peptide into the cytoplasm, but the presence of the L2 CPP adjacent to the retromer binding sites provides a simple solution to this problem. We synthesized a short peptide containing the HPV16 L2 retromer binding sites, the HPV16 or HPV31 L2 CPP, and flanking sequences. When this peptide is added to the culture medium, the peptide enters cells, binds retromer and displaces it from incoming virus, and aborts infection by several pathogenic HPV types [60]. As predicted, inhibitory activity is dependent on both the retromer binding sites and the CPP on the peptide. The inhibited virus initially accumulates in the endosome, as is the case for other manipulations that directly prevent retromer-L2 binding, but following peptide treatment, the virus is eventually eliminated from cells. The peptide also inhibits HPV16 pseudovirus infection in a short-term cervicovaginal infection model in mice, demonstrating that active peptide can enter keratinocytes and inhibit infection in intact tissue. This approach is possible because both the retromer binding sites and the CPP are short linear amino acid sequences that do not require the rest of the L2 protein or the intact capsid structure to function. Although these peptides may not emerge as clinically useful agents, these experiments provide important proof-of-principle that intracellular virus trafficking is a potential therapeutic vulnerability warranting the development of more drug-like molecules. More generally, these experiments established the feasibility of disrupting important cytoplasmic protein–protein interactions with soluble peptides with cell-penetrating activity. Finally, a lipopeptide derived from the N terminus of L2 displays potent activity against HPV entry, but its mechanism of action is not known [61].

The genetic and biochemical analysis of retromer action during HPV entry also suggests that compounds that inhibit Rab7, TBC1D5, or retromer itself may have activity against HPV. Because retromer evolved to transport cellular, not viral, proteins, compounds that block retromer action are also likely to inhibit transport of cellular cargos. In fact, trafficking of cellular cargos is affected by the peptides discussed above as well as by expression of dominant-negative Rab7 (unpublished and [60]). However, these manipulations do not cause any obvious cellular toxicity under conditions where HPV is strongly inhibited. This finding implies that HPV entry is strictly dependent on retromer and Rab7, while cells may be able to use alternative mechanisms to support normal cellular function. Importantly, trafficking of cellular cargos can still proceed in cells knocked down for TBC1D5, reflecting a unique HPV vulnerability and suggesting that agents that specifically target Rab7 cycling may inhibit HPV trafficking while sparing cellular cargos. γ-secretase inhibitors are also predicted to be potent anti-viral agents, but their utility may be limited by side effects due to inhibition of the proteolytic action of γ-secretase on cellular substrates. Development of compounds that specifically inhibit the membrane chaperone but not the proteolytic activity of γ-secretase may provide useful anti-HPV agents. It should be noted that, like vaccination, the primary benefit of HPV entry inhibitors would be to prevent spread of the virus from one individual to another.

## 7. Conclusions

Recent study of HPV entry and trafficking and the elaboration of the retrograde model of HPV entry have revolutionized our understanding of this process and suggested novel preventive approaches. Although the broad outlines and some of the fascinating details of this process have emerged, there is still much to learn. Further study of HPV entry will reveal additional interesting aspects of this complex process and may lead to the development of clinically viable strategies to combat HPV infection. These studies will also continue to provide important new insights into fundamental cellular processes such as the action of retromer and CPPs and other aspects of intracellular protein trafficking. As has historically been the case, viruses will serve as valuable tools to understand biology.

## 8. Patents

Pengwei Zhang and Daniel DiMaio are inventors on a patent application related to this work.
